# The Inhibiting Effects of High-Dose Biochar Application on Soil Microbial Metagenomics and Rice (*Oryza sativa* L.) Production

**DOI:** 10.3390/ijms242015043

**Published:** 2023-10-10

**Authors:** Nanyan Zhu, Qiaoqiao Yu, Lingqi Song, Haijun Sheng

**Affiliations:** 1 College of Animal Science and Technology, Yangzhou University, Yangzhou 225000, China; dx120200148@stu.yzu.edu.cn; 2 Jiangsu Key Laboratory of Crop Genetics and Physiology, Agricultural College of Yangzhou University, Yangzhou 225000, China; 15046086520@163.com; 3 College of Environmental Science and Engineering, Yangzhou University, Yangzhou 225000, China; slq231010@163.com

**Keywords:** rice, biochar, peanut waste, soil microbial metagenomics, nutrient cycle

## Abstract

Biochar is usually considered as an organic improver which can improve soil and increase crop yields. However, the unrestricted application of biochar to normal-fertility farmland will cause chemical stress on crops and affect agricultural production. At present, the effects and mechanisms of high-dose applications of biochar on rice (*Oryza sativa* L.) production and soil biological characteristics have not been fully studied. In this greenhouse pot experiment, combined with soil microbial metagenomics, three treatments in triplicates were conducted to explore the responses of rice production, soil chemical properties, and soil biological properties to high-dose applications of biochar (5%, *w*/*w*) prepared using peanut waste (peanut hulls and straw). The results show that peanut hulls, with a loose texture and pore structure, are a raw material with stronger effects for preparing biochar than peanut straw in terms of its physical structure. In a rice monoculture system, high-dose applications of biochar (5%, *w*/*w*) can slightly increase the grains per spike, while significantly inhibiting the spike number per pot and the percentage of setting. High-dose applications of biochar also have significant negative effects on the diversity and stability of soil bacterial and archaeal communities. Moreover, the microbial metabolism and nutrient cycling processes are also significantly affected by changing the soil carbon/nitrogen ratio. This study discusses the response mechanisms of rice production and soil biology to high-dose biochar applications, and complements the understanding of irrational biochar application on agricultural production and land sustainability.

## 1. Introduction

The excessive use of inorganic fertilizer and the pursuit of a high yield of crops in traditional intensive production cannot meet the requirements of modern agriculture for sustainable development. One-third of the soil around the world today has poor conditions, including single production functions and ecological pressures (such as degraded soil, reduced fertility, overloaded production, eroded soil, and unsustainable management methods) [1]. Biochar is considered as an effective material to improve soil fertility, regulate soil acid alkalinity, and improve planting efficiency [2]. However, a series of cases have shown that the application of biochar alone or in excess carries the risk of reducing crop yields [3,4,5].

Biochar, a solid byproduct with rich carbon, is produced through biomass pyrolysis under oxygen-limiting conditions [6]. Its raw materials can be various biomasses, including agricultural and forestry waste (crop straw, fruit hulls, wood, etc.) and organic matter (OM), such as bones and feces [7]. The physicochemical properties of biochar (specific surface area, surface functional groups, pH, ash content, carbon/nitrogen ratio (C/N), etc.) are affected by differences in preparation processes, such as raw materials, preparation methods, reaction temperature, and reaction time [8]. Considering cost and storage, lignocellulose is an ideal raw material, which is represented by agricultural and forestry waste, such as crop straw, plant roots, stem hulls, and sawdust [9,10]. Since biochar is mainly composed of organic carbon, single applications of biochar cannot fully provide plenty elements (nitrogen, phosphorus, potassium, etc.) for crop growth, and may have a risk of yield reduction, especially in oligotrophic soil. Therefore, biochar taken as an organic modifier is always combined with other fertilizers in most studies [3]. Biochar, which can reduce stubble burning, pesticide use, and N_2_O and CH_4_ emissions from agriculture (especially rice fields), as well as being able to absorb harmful substances (such as heavy metals), has become one of the important research and promotion directions of agriculture [11,12]. Peanut hulls and straw are agricultural wastes that are formed during the peanut production process. According to statistics, China produces up to 4.5 million tons of peanut hulls and approximately 30 million tons of peanut straw annually [13]. Peanut hulls have abundant pore structures on the surface and loose internal textures, making them a good precursor for developing high-performance adsorption materials. At present, they are used to prepare biochar by some scholars for studying their role in improving the retention of nitrogen in soil as a nutrient additive of tobacco plants [14], or in removing heavy metal ions [15].

Soil microorganisms with various categories and large quantities are an important part of farmland ecosystems, mainly including bacteria, fungi, and archaea. They participate in the decomposition and transformation of OM and soil nutrients through their own growth and metabolism, providing power and guarantees for soil nutrient circulation and energy flow [16]. Therefore, soil microbial communities are very sensitive to environmental changes. Field fertilizer management can directly or indirectly change the nutrient balance of farmland ecosystems by changing the nutrient supply status, thereby affecting the biological characteristics of soil [17]. Soil biological characteristics (microbial biomass, community structure, etc.), which can reflect the quality and fertility of soil, are considered as important indicators of soil environmental quality evaluation [18]. Many studies have discussed the response of farmland ecosystems to biochar addition from the perspective of crop physiology and soil biological communities [19,20]. It has been confirmed that low concentrations of biochar can promote crop yield and maintain the stability of soil microbial communities. However, high doses of biochar (3%, *w*/*w*) application causes toxicity (chemical stress) to crop growth and soil microorganisms [4], and both the diversity indexes and metabolic function of soil microorganisms tend to decrease [5]. Soil archaeal communities play a key role in the C- and N-cycling of soil participates in some processes (CH_4_ metabolism, ammonia oxidation, etc.), which is a potential nutrient pool in soil [21]. However, previous studies on soil microbial communities under different biochar application modes have mostly focused on bacterial and fungal communities [22]; research on soil archaea is still limited [23,24].

With the deepening of relevant research on soil microorganisms, research methods are constantly improving. At present, the research methods and techniques of soil microbial community structures at home and abroad are mostly focused on phospholipid fatty acid analysis [25], quantitative polymerase chain reaction (PCR) amplification techniques, and high-throughput sequencing techniques [26]. The metagenome sequencing technique is a new perspective to analyze the changes of microbial community structures. It can span the process of microbial isolation, purification, and culture, reveal the genetic composition and community function of all microorganisms existing in the environment, and analyze the relationship between soil microbial species and the environment [27]. It has been found that this technique can be used to simultaneously sequence and analyze soil bacterial, fungal, and archaeal communities, so as to further explore the differences in soil microbial community compositions and structures under various field management practices. Compared with that of soil bacteria, the number of archaea is quite low. However, archaeal communities play a vital role in cycle and metabolism of carbon and nitrogen [28], and the response of archaeal communities to environmental changes is an important supplement to the study of soil microorganisms.

It is of great significance for fertilizer management in paddy fields to clarify the effects of biochar from different raw materials on rice production and soil biological characteristics. In this study, combined with soil microbial metagenomics and analysis techniques, the effects of field fertilization management modes of high-dose peanut waste (peanut shells and straw) biochar application on rice production and yield composition were studied in a greenhouse pot experiment, and the interaction relationships between rice production, soil chemical properties, and soil biological characteristics were also explored, which provided theoretical support for the standardized application of bio-waste biochar in agricultural soil improvement, energy conservation, and emission reduction.

## 2. Results

### 2.1. Effects of High-Dose Biochar Application on Soil Properties

The general chemical properties of all experimental soil samples are summarized in Table 1. It was shown that the soil pH increased significantly (*p* < 0.05) with biochar application, and the contribution of PSBC (peanut straw biochar) to the soil pH was more obvious than that of PBC (peanut hull biochar), which was closely related to the pH value of the biochar itself. The content of soil NO_3_^−^–N (nitrate nitrogen) decreased notably under PBC and PSBC application (*p* < 0.05). Moreover, both the AK (available potassium) and OM (organic matter) levels were enhanced considerably relative to the CK (control).

### 2.2. Effects of High-Dose Biochar Application on Rice Yield and Yield Components

Rice yield is composed of four important factors: the spike number per pot, the grain number per spike, the thousand-grain weight (TGW), and the percentage of setting (SP). High-dose biochar application increased the grains slightly, but not to a significant level (*p* > 0.05). However, it was observed that other parameters related to rice yield were reduced relative to the CK.

### 2.3. Effects of High-Dose Biochar Application on Structure and Composition of Soil Microorganisms

#### 2.3.1. Quality Evaluation of Metagenomic Sequences

Base quality and distribution maps of the original sequences (Figure S1) showed that the contents of base G–C and A–T were equal, and remained stable throughout the entire sequencing process with a horizontal line. After quality control, different samples were processed to obtain a total sequence number of 0.97–1.12 million. The average length of each sample was over 500 bp (Table S1). This indicated that the construction quality of the sample library was relatively high, and the sequencing quality was good, which met the requirements for subsequent analysis.

#### 2.3.2. α Diversity Analysis of Soil Microorganisms

**Figure 1:**
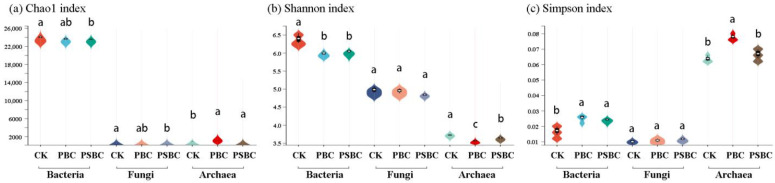
(**a**) Chao 1; (**b**) Shannon; (**c**) Simpson diversity index of soil bacterial, fungal, and archaeal communities. CK: control; PBC: peanut hull biochar; PSBC: peanut straw biochar. Different lowercase letters indicate significant differences between various treatments based on a one-way ANOVA followed by Duncan’s multiple-range tests (*p* < 0.05).

Microbial diversity is closely related to soil ecosystem function and environmental stability. A high level of microbial diversity is an important guarantee for resisting external environmental interference [29]. The *α* diversity values of the soil microbial communities were estimated using the Chao 1, Shannon, and Simpson indices in this study (Figure 1). The Chao 1 index is an important indicator that reflects the abundance of microbial communities; the Shannon and Simpson indices reflect the diversity of microbial communities. Usually, the larger the Chao 1 and Shannon indices, the smaller the Simpson index, indicating that the sample contains more species, and that the *α* diversity is higher. The results showed that the interaction of high-dose biochar (PBC and PSBC) applications and the soil environment significantly reduced the *α* diversity of the soil bacterial and archaeal communities, but it had no noticeable influence on the soil fungal communities (*p* > 0.05). Compared to the PSBC treatment, the PBC had a stronger influence on the stability of soil microbial communities (Figure 1).

#### 2.3.3. Differences in Structure and Composition of Soil Microorganisms

Quantitative statistics of the rice soil microorganisms were carried out under the different treatments (Table S2). The microbial species of nine soil samples were distributed in 12 phyla and 224 classes. Bacteria, fungi, and archaea from the three treatments were extracted, and databases were built for subsequent analysis. The Venn diagrams (Figure S2) reflect the similarity and overlap of the microbial community composition in each sample. It was shown that the number of unique microbial species in the three groups of samples was lower than that of the common species, and this number in the CK was slightly higher than that for the PBC and PSBC.

**Figure 2:**
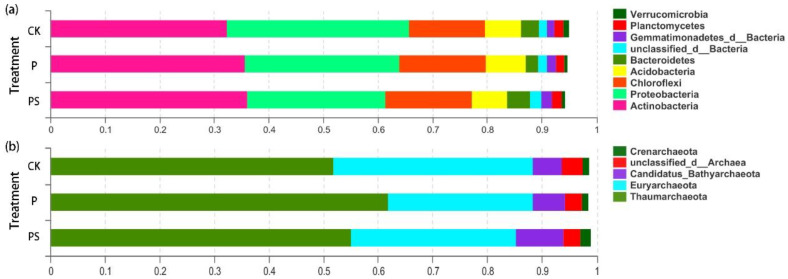
Effect of biochar application on the relative abundance of soil (**a**) bacterial and (**b**) archaeal phyla. Only the phyla with RPKM ≥ 1% are presented in this figure.

The soil bacterial abundant phyla (Figure 2) and genera (Figure S3) with RPKM ≥ 1% were selected for observation. The dominant strains of each treatment were similar, but there were differences in the relative abundance of strains. The co-application of fertilizer with biochar increased the dominant microorganisms compared to that with the control. The relative abundance of the top nine most abundant phyla in the soil bacterial communities accounted for 99.50% in the CK, 95.33% for the PBC, and 95.19% for the PSBC, respectively. In addition, 34.24%, 30.8%, and 33.90% of *p_Actinobacteria* in the bacterial phyla were observed in the PSBC, CK, and PBC groups, respectively (Figure 2a). The total relative abundance of the top five abundant phyla in the archaeal communities accounted for 99% of all these three samples. The proportion of *p_Thaumarchaeota* increased from 51.81% in the CK to 61.82% with the PBC and 55.05% with the PSBC (Figure 2b). Circos diagrams (Figure S3) reflected the dominant genera and the proportion of different treatments. Among the relative abundance of the top ten bacterial genera, only *g_Nocardioides* and *g_unclassified_c_Betaproteobacteria* were dominant in the CK. The relative abundance of the top six archaeal genera were relatively lower in the CK treatment compared to that with the PBC and PSBC.

**Figure 3:**
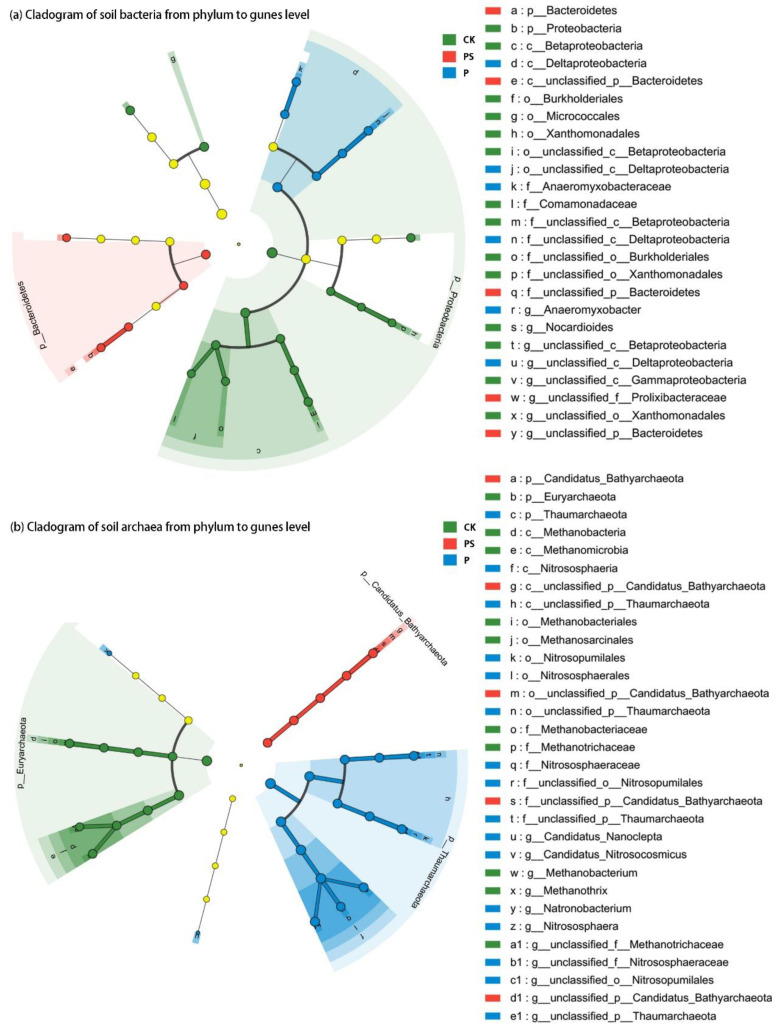
Cladogram of soil (**a**) bacterial and (**b**) archaeal communities in all of the treatments. The taxa with significantly different abundances among treatments are represented by colored dots, and from the center outward, they represent the phylum, class, order, family, and genus levels. The colored dots represent the microbial groups that are significantly enriched in the corresponding group. The colored shadows represent trends of the significantly differed taxa.

Linear discriminant analysis effect size (LEfSe) was adopted to determine the biomarkers that could best explain the differences between the treatments, and to visualize the influencing degree and evolution law of these biomarkers (Figure 3 and Figure S4). The comparison strategy was set to be all-against-all (only species with differences in multiple groups could be considered as different biomarkers), and the threshold was set to be 2.0 (Table S3). However, a threshold of 3.5 was set for a clearer display in Figure 3. The results showed that there were clear and definite differences and hierarchical relationships in the biomarkers of the bacterial and archaeal communities among the three treatments. Compared with the CK and PBC treatments, the number of biomarkers for the PSBC was the lowest. Biochar application significantly reduced (LDA > 4.5) the relative abundance of two main phyla (*p_Proteobacteria* and *p_Euryarchaeota)* in the soil microbial communities (Figure S4).

#### 2.3.4. β Diversity Analysis of Soil Microorganisms

**Figure 4:**
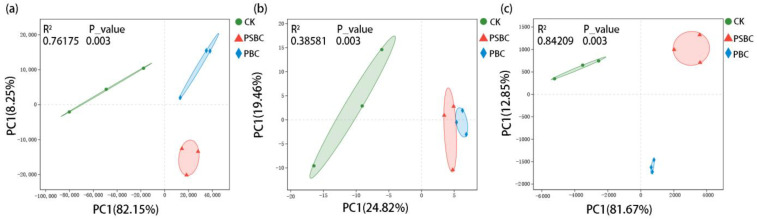
PCA of soil (**a**) bacterial, (**b**) fungal, and (**c**) archaeal communities at the species level and PERMANOVA at 99% level based on Bray–Curtis distance in the various treatment.

A principal component analysis (PCA) was carried out on the three treated soil microorganisms at the species level. The *p*_values of PERMANOVA were all less than 0.05, which indicates that the reliability of the sample analysis was high (Figure 4). The results showed that samples in different groups represent obvious intra-group aggregation and inter-group dispersion. In the PCA results of the soil fungal communities, the R^2^ and the explanation of the PC1-axis were significantly lower than those of the bacterial and archaeal communities, indicating that different biochar applications have stronger influences on soil bacterial and archaea communities than on fungal communities.

### 2.4. Relationships between Soil Microorganisms, Environmental Factors, and Rice Yield

**Figure 5:**
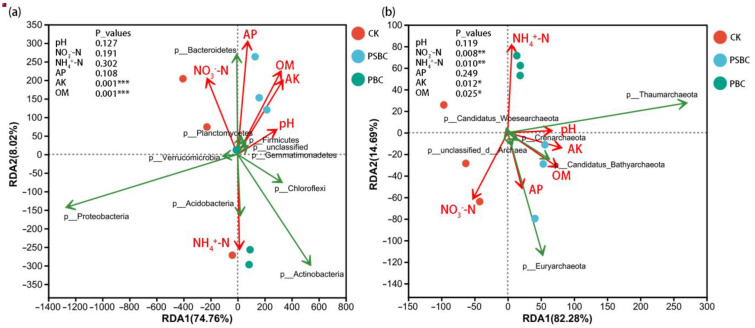
RDA of soil (**a**) bacterial and (**b**) archaeal phyla with soil chemical properties. The soil chemical properties were fitted to the ordination plots using a 999-permutation test (*p*_values). NO_3_^−^–N: nitrate nitrogen; NH_4_^+^–N: ammonium nitrogen; AP: available phosphorus; AK: available potassium; OM: soil organic matter. Asterisks denote the significance level: * *p* < 0.05, ** *p* < 0.01, *** *p* < 0.001.

Redundancy analysis (RDA) was used to quantify the effects of environmental chemical properties of rice soil on the bacterial and archaeal communities at the phylum level (Figure 5). Among all the soil chemical properties, compared with that of other factors, the effect of NH_4_^+^–N on both the bacterial and archaeal communities showed an opposite trend. The effects of the soil AP, NO_3_^−^–N, NH_4_^+^–N, and OM on the bacterial community structures were all represented by long arrows, indicating that they were important soil environmental factors affecting the composition of bacterial communities at the phylum level (Figure 5a). AP, NO_3_–N, and OM were all negatively correlated with some bacterial phyla (*p_Proteobacteria*, *p_Actinobacteria*, *p_Chloroflexi*, etc.), while NH_4_^+^–N was positively correlated with them. While some bacterial phyla (*p_Bacteroidetes*, *p_Firmicutes*, etc.) had an inhibitory relationship with them, tending towards an environment of high AP and OM; low NH_4_^+^–N. NO_3_^−^–N and NH_4_^+^–N are the two main soil environmental factors that affect the composition of archaeal communities (*p* < 0.01) (Figure 5b). Some archaeal phyla (*p_Candidatus_Bathyarchaeota*, *p_Euryarchaeota*, *p_Crenarchaeota*, etc.) tended towards an environment of high contents of soil NO_3_^−^–N and AP, and low NH_4_^+^–N.

**Figure 6:**
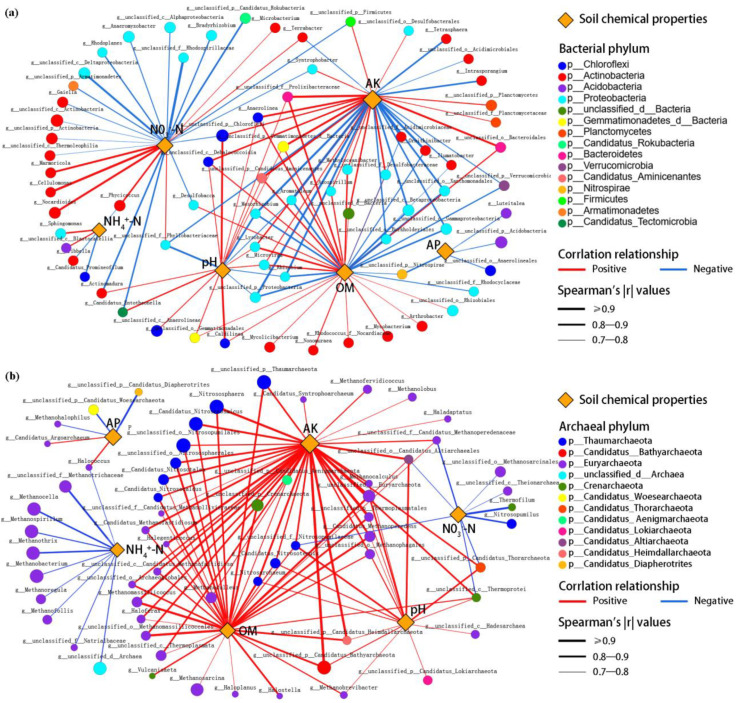
Correlation network between soil (**a**) bacterial and (**b**) archaeal genera and soil chemical properties based on Gephi 0.9.2 software. Network node properties and the Spearman’s correlation values of each node are shown in Table S4.

The correlation network between the microbial genera and the environmental factors was further constructed by calculating the correlation coefficient between the soil microorganisms and the soil chemical properties. In addition, the possible interaction relationships between the structure of the microbial communities and the soil chemical properties at the genus level were analyzed. It showed that AK and OM were the two key soil environmental factors affecting the structure of microbial communities (Figure 6). At the same time, NH_4_^+^–N and pH, as important soil factors, had an influence on the bacterial community structure at the genus level. *g_Intrasporangium*, *g_Tetrasphaera*, *g_Ornithinibacter*, etc., in *p_Actinobacteria* showed a significant negative correlation with AK. *G_Nonomuraea*, *g_Mycobacterium*, *g_Rhodococcus_f_Nocardiacea*, etc., represented a significant positive correlation with OM. Most genera (*g_unclassified_c_Deltaproteobacteria*, *g_unclassified_f_Rhodospirillaceae*, *g_unclassified_c_Betaproteobacteria*, *g_Aromatoleum*, etc.) in *p_Proteobacteria* exhibited strong negative correlations with soil chemical properties (Figure 6a, Table S4a). Based on the correlation network analysis of archaea and environmental factors, it can be seen that *p_Thaumarchaeota*, *p_Euryarchaeota*, *p_Candidatus_Bathyarchaeota, p_Crenarchaeota*, etc., showed a clear preference for an environment of high AK, OM, and pH, and low NH_4_^+^–N and NO_3_^−^–N (Figure 6b, Table S4b).

**Figure 7:**
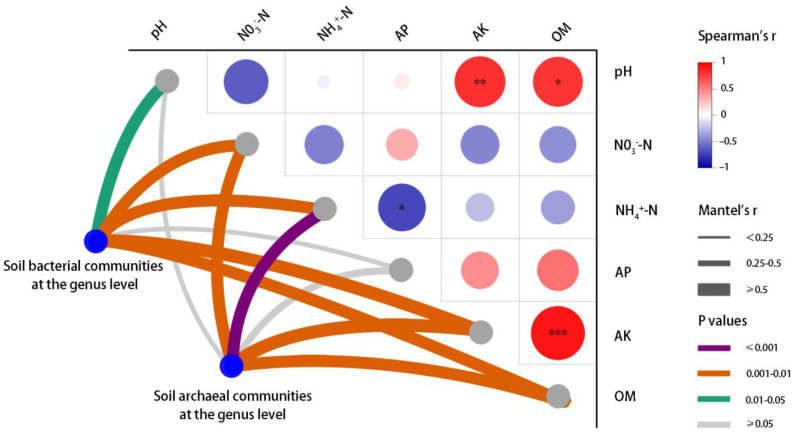
Pairwise comparisons of soil chemical properties and rice yield. TGW: 1000-grain weight; SP: percentage of setting. Mantel tests depicted the association of soil bacterial and archaeal phyla with soil variables, and the width of each edge matched Mantel’s r value. Asterisks denote the significance level: * *p* < 0.05, ** *p* < 0.01, *** *p* < 0.001.

Spearman correlation analysis and a Mantel test was used to explore the possible effects of the interaction between soil factors and microorganisms on rice yield (Figure 7). AK and OM were positively correlated with the number of grains. SP was significantly correlated with NH_4_^+^–N (negative correlation) and NO_3_^−^–N (positive correlation). The results of the Mantel tests showed that there were strong correlations between the microbial communities and the soil environmental factors at the phylum level, which also verified the reliability of the RDA (Table S5).

To further explore the indirect effect of soil microorganisms on rice yield, the genera that were mined in the LEfSe (Table S3) and significantly correlated with soil chemical properties (Table S4) were selected and combined with rice yield components to conduct a linear regression analysis. It was found that a total of seven bacterial genera and one archaea genus that were screened were significantly correlated with the rice yield (Table S6). Among them, one archaeal genus (*g_Methanothrix*) and four bacterial genera (*g_unclassified_c_Betaproteobacteria*, *g_Nocardioides*, *g_Phycicoccus*, *g_Marmoricola*) were significantly enriched in the CK; three bacterial genera (*g_unclassified_c_Deltaproteobacteria*, *g_Anaeromyxobacter*, *g_unclassified_p_Candidatus_Rokubacteria*) which had significant negative correlations with the soil NO_3_^−^–N were significantly enriched by the PBC. There were five bacterial genera that had significant correlations (*p* < 0.05) with the spikes, TGW, and SP (Table S4). *g_unclassified_c_Betaproteobacteria* was negatively correlated with the soil AK and OM, and was the only genus which had a positive correlation with grains. Other screened genera were all involved in N-cycling.

### 2.5. Effect of High-Dose Biochar Application on Soil Microbial Metabolism

**Figure 8:**
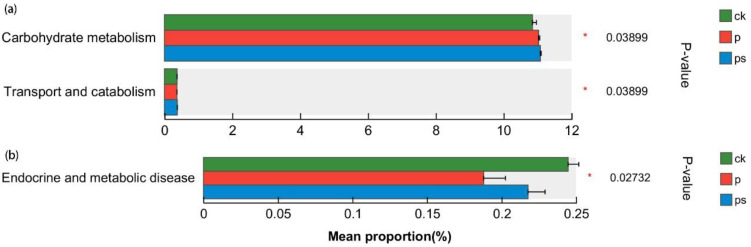
Kruskal–Wallis H test bar plot of soil (**a**) bacterial and (**b**) archaeal metabolism at KEGG_L2 level. Asterisks indicate significant differences between various treatments based on Kruskal-Wallis H tests (*p* < 0.05).

The KEGG functional information of each sample was analyzed to predict the effect of biochar application on the functions of soil microorganisms. The relative abundance of protein sequences of each sample was statistically analyzed at the KEGG_L1 level. Most protein sequences (total > 80%) were annotated to three functions: metabolism, genetic information process, and environmental information process (Figure S5). Comparative analysis at the KEGG_L2 level showed that the significantly differential functions in bacteria were the carbohydrate metabolism (*ko00620*, *ko00020*, *ko00640*, *ko00053*, *ko00030*) and transport and catabolism (*ko04145*, *ko04137*) (Figure 8 and Figure S6). The significant differential function in archaea was endocrine and metabolic disease, which was concentrated in the K-ion metabolism function (*ko04931*, *ko04932*).

## 3. Discussion

### 3.1. Effects of High-Dose Biochar Application on Soil Fungal Communities

It has been mentioned in many rice-related studies that different treatments can affect the abundance and structure of soil fungi communities. The interactions between soil fungi and some aspects (yield, biomass, root differentiation mechanisms, etc.) are also explored [3,22]. However, bacterial communities are more susceptible to environmental factors compared to fungi [30]. The main reason for affecting the structure of soil fungal communities is plant fungal diseases, especially the soil-borne fungal diseases that exist in the form of fungal nuclei or hyphae [31]. In this study, compared with bacteria and archaea communities, it was also found that different treatments had a smaller influence on the number of soil fungi (Table S2) and the compositional similarity (Figure 3, Figures S2 and S7), and did not significantly (*p* > 0.05) affect the *α* diversity (Figure 1) of the microorganisms. The PCA of the fungal phyla showed that the interpretation of the PC1-axis was only 24.82%, and there were some overlaps in the fungal communities between the PBC and PSBC treatments (Figure 5b). It can be considered that the biochar application had a relatively small influence on the structure of soil fungal communities in this study. Therefore, similarities and differences in the structure and metabolic functions of soil bacterial and archaeal communities would be further analyzed. The interaction between soil chemical properties and soil bacterial and archaeal communities would also be explored under high-dose biochar application.

### 3.2. Effects of High-Dose Biochar Application on Soil Bacterial and Archaeal Communities

Soil physical and chemical properties directly affect the nutrient supply capacity and survival environment of soil microorganisms. Therefore, soil microbial communities are very sensitive to changes in the soil environment [28]. Some studies have pointed out that low-dose biochar application has a positive influence on soil microbial communities, but high-dose biochar application produces chemical stress on soil microorganisms [19]. Similar results were also found in this study. Although high-dose biochar application did not change the structural composition and dominant phyla and genera, it affected the relative abundance (Figure 2 and Figure S3) and significantly reduced the *α* diversity of the soil bacterial and archaeal communities (Figure 1). *p_Actinobacteria* is a microbial community with the highest relative abundance and a strong stress tolerance amongst soil bacterial communities [32,33,34]. In this study, the relative abundance of *p_Actinobacteria* in the CK was significantly higher than that with PBC and PSBC (Figure S4). This indicated that *p_Actinobacteria* was obviously more adaptable to the adverse soil environment with high-dose biochar applications than other microbial communities.

### 3.3. Interaction between High-Dose Biochar with Rice Production and Soil Microorganisms

The RDA results indicated that there were significant symbiotic or inhibitory relationships between some genera of bacterial phyla (*p_Proteobacteria*, *p_Actinobacteria*, *p_Chloroflexim*, *p_Bacteroidetes*, *p_Firmicutes*, etc.) and archaeal phyla (*p_Euryarchaeota*, *p_Candidatus_Bathyarchaeota*, *p_Crenarchaeot*, etc.) (Figure 5). The Mantel tests were carried out to further validate that the content of the soil AK, OM, and inorganic-N could be identified as key soil environmental factors that altered the structure of the bacterial and archaeal communities in this study (Figure 7). The study of Aller and Kemp also reached a similar conclusion that the pH and carbon/nitrogen ratio (C/N) were key soil factors affecting the archaeal community structure [35].

It is worth noting that the soil NO_3_^−^–N content was significantly correlated with many bacterial genera in *p_Actinobacteria* and *p_Proteobacteria*, and there was a significant negative correlation between the soil NH_4_^+^–N content and many archaeal genera in *p_Euryarchaeota* (Figure 6). It is indicated that these genera may be irreplaceable in the soil N-cycle or have strong responses to the content of soil inorganic-N [36]. In this study, subgroups of ammonia-oxidizing bacteria (AOB) and ammonia-oxidizing archaea (AOA) also showed obvious characteristics of ecological niche differentiation. *p_Proteobacteria* and *p_Thaumarchaeota* were the dominant groups of AOB and AOA, respectively. The relative abundance of *c_Betaproteobacteria* subgroups (*f_Intrasporangiaceae*, *f_Comamonadaceae*, etc.) in the CK was higher, while the application of biochar significantly increased the relative abundance of *p_Thaumarchaeota* (*o_Nitrosopumilales*, *o_Nitrososphaerales*, etc.) in the PBC sample (Table S3). The application of biochar can promote the emission of CO_2_ and create a hypoxic environment [17]. It may inhibit the activity of AOB, which is also one of the reasons that affect the ecological niche differentiation of AOB and AOA subgroups in this study [28,37]. Some studies have proposed that the denitrification process competes with the dissimilatory nitrate reduction to ammonium (DNRA) for NO_3_^−^–N in the environment. Under high C/N conditions, DNRA dominates the competition for NO_3_^−^–N in the environment; when the carbon source supply is insufficient, NO_3_^−^–N is mainly removed through the denitrification process [38,39]. In this study, some denitrifying bacteria (*f_Flavobacteriaceae*, *f_Cytophagaceae*, *f_Zoogloeaceae*, etc.) were significantly enriched in the CK, while biochar application improved the relative abundance of some microorganisms (*f_Anaeromyxobacteraceae*, *f_Planctomycetaceae*, etc.) related to DNRA (Table S4) [40]. It is proven that biochar application can increase the C/N in soil by applying the exogenous organic C, stimulate the occurrence of DNRA [41], and reduce highly mobile NO_3_^−^–N to more stable NH_4_^+^–N, thereby reducing the N-loss and improving the soil N-use efficiency [42]. Moreover, the higher specific surface area with PBC (Table 2) means more surface charges, which can better serve as an “electron donor” to promote DNRA [41]. To a certain extent, this also explains the differences in NH_4_^+^–N content among the various treatments in this study.

Furthermore, effects of the soil AK, OM, and pH on the microbial communities were relatively independent of that of soil inorganic-N in this study (Figure 7). The main reason may be that biochar affects the community structure of soil microorganisms by regulating the nutrient cycle of nitrogen and carbon. NO_3_^−^–N as an available nutrient can be directly utilized by microorganisms to participate in the soil nitrogen metabolism process; organic-C, as the external reflection of soil carbon sources, mediates the C-cycling process to affect the activities of soil microorganisms [43]. It can be considered that the biochar application directly controls the C/N by changing the supply of bioavailable carbon and nitrogen in soil. By regulating the content of organic-C and inorganic-N, the community structure of soil microorganisms related to the N-cycle and OM decomposition is affected, thereby mediating the metabolic processes of key microorganisms.

### 3.4. Interaction between High-Dose Biochar Application and Microbial Metabolic Functions

High-dose biochar application can affect the metabolic processes of polysaccharides in carbohydrates by increasing the activity of enzymes involved in the hydrolysis process of polysaccharides, and then regulate the microbial community structure through metabolites [44]. In this study, biochar application provided a large amount of organic-C, and the main performance of the effect on the functions of the soil microbial communities was the increase in the relative abundance of carbohydrate metabolism-related protein products. This may be due to the fact that the abundant potassium in biochar has a catalytic effect on metabolic processes, such as the decomposition and transfer of carbohydrates, which can promote the synthesis of related enzymes and improve their activities [45]. At the KEGG_L2 level, the relative abundance of related genes increased significantly in the two treatments with biochar application (Figure 8). The results of this study showed that biochar application promoted the carbohydrate metabolism in general, and affected the carbon metabolism process of soil microbial communities by regulating the synthesis of key protein sequences controlling the synthesis of related enzymes.

The phosphorus cycle is also an important part of the nutrient cycle. Studies have confirmed that the addition of organic-C has a significant effect on the phosphorus cycle process during the rice heading stage [46]. In this study, biochar application increased the supply and retention of AP in the soil (Table 1), but the bacterial and archaeal communities had little response to the change in the soil AP (Figure 6), and the relative abundance of functional protein sequences related to the phosphorus metabolism did not change significantly (Figure 8). This may be due to the dynamic change in the rice demand with the PBC during the whole growth period [47].

### 3.5. Possible Inhibiting Mechanism of High-Dose Biochar Application on Rice Production

The results of this pot experiment showed that, under the condition of normal basic soil productivity, compared with that of inorganic fertilizer (CK), the application of high-dose biochar (5%, *w*/*w*) produced with peanut hulls (PBC) or peanut straw (PSBC) significantly reduced the spike and TGW indicators, which would lead to the reduction of rice yield by more than 20%.

It is speculated that there are several possible reasons for this phenomenon. Firstly, the alkalinity of the biochar directly increased the soil pH, which was significantly correlated with the decrease in the panicle number (Figure 7). Secondly, the adsorption and fixation of inorganic-N in soil via biochar application is the reason for the down-regulation of nitrification [1]. In this study, the biochar application may have reduced the ammoxidation and nitrification potential of the soil microorganisms, resulting in a increase in the soil NH_4_^+^–N content and a decrease in NO_3_^−^–N. This would be significantly correlated with the decrease in the spike, TGW, and SP indicators, which jointly affected the rice yield. Thirdly, biochar with high organic-C can intensively enhance the C/N of soil [48], stimulate and enhance DNRA, and grab soil NO_3_^−^–N, which may also be one of the reasons for the decrease in the soil nitrification process in this study. In addition, the application of biochar can strengthen the enrichment effect of cation ions (P^+^, K^+^), delay the release of nutrients, and increase the retention of nutrients in the soil [49]. However, in this study, the controlled release of nutrients through high-dose biochar addition may not be friendly to plants in the early growth period of rice.

Under the same level of inorganic-N supply, the content changes in the soil nutrients (Table 1) and the reduction in yield (Table 3) with the PBC treatment are more significant than those with the PSBC, which may be due to the higher specific surface area (SSA) of the PBC (Table 2). The greater surface charges provided by the higher SSA will enhance DNRA and lead to competition for the soil NO_3_^−^–N with the nitrification process. Furthermore, biochar with a high SSA can recruit more N_2_O-reducing bacteria to enhance denitrification and reduce N-loss [50].

### 3.6. Applicability and Limitations

Unlike the field experiment, this greenhouse pot experiment is more precise in controlling environmental variables, reducing the interference of external environmental factors. In exploring the inhibiting effect of high-dose biochar on rice production and the interaction mechanism with soil microbial communities, the experimental results have a higher reliability. However, the results still need to be supported by different concentration gradients of biochar application experiments and field experiments. These results will be presented in a future series of reports.

Additionally, considering the porosity differences of different biochar, the lack of changes in the soil porosity before and after biochar application is one of the deficiencies of this study [20]. Different soil environments not only directly affect the structure of soil microbial communities, but also have regulatory effects on the growth and nutrient uptake of plant roots [51]. In the future, it is necessary to further explore changes in soil porosity and the response of plant roots to various biochar applications.

From the perspective of agricultural practice management, the cost of the large-scale preparation, distribution, and application of biochar is very high. A major obstacle faced by companies and farmers is the economic feasibility of the biochar market. In the future, a green and efficient application mode of low-dose biochar combined with organic or controlled-release fertilizer can be considered to reduce the economic constraints of biochar in large-scale practical agricultural application.

## 4. Materials and Methods

### 4.1. Experimental Site, Materials, and Treatments

This experiment was performed in a greenhouse at an experimental base (32°24′ N, 119°26′ E) at the College of Environmental Science and Engineering of Yangzhou University. The experimental site is located in Yangzhou City, Jiangsu Province. The annual average temperature, rainfall, and sunshine hours in this region are 17.5 °C, 1020 mm, and 1824 h, respectively. The greenhouse environment was simulated as a field condition, of which the average temperature was 29.8 °C and the relative humidity was 45–70% during the experiment.

**Figure 9:**
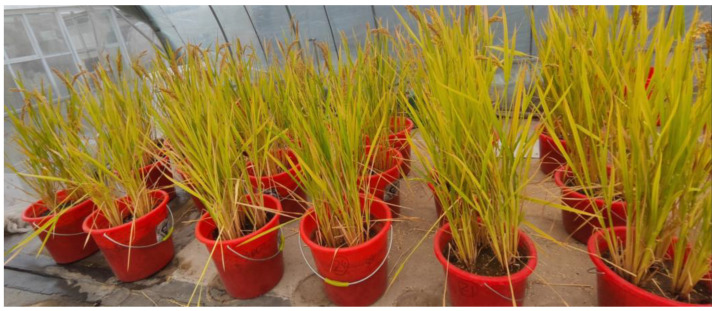
Experimental site and materials of this greenhouse pot experiment.

In this experiment, a single-factor design was adopted, and three treatments were set up, including compound fertilizer (control (CK)), compound fertilizer + peanut hull biochar (PBC) and compound fertilizer + peanut straw biochar (PSBC). Each treatment was repeated 6 times, and a completely randomized design was used with a total of 18 pots. The experimental soil was local silt loam, and the basic soil productivity of the topsoil (0–20 cm) was evaluated before sowing (Table 2). Impurities (stones, plant residues, etc.) were picked out in the experimental soil. The soil was screened with a 10-mesh soil sieve and stored at 4 °C for standby. After drying, the soil was sieved to 2 mm and mixed thoroughly; 10 kg per pot of soil was weighed and put into plastic buckets with a size of 30 × 25 × 25 cm (height × width × length) and no holes in the bottom. Each pot was separated by 20–30 cm as the edge area (Figure 9).

The experimental rice (*Oryza sativa* L.) variety (Nangeng-46) that is widely planted in the local area was selected for this study. Rice seedlings were transplanted into pre-prepared pots. There were 3 holes in each pot and 3 plants, without tillering, in each hole. Peanut hulls and straw pyrolyzed at 500–600 °C were used as raw materials to produce the biochar, which were purchased from Zhironglian Technology Co., Ltd. (Nanjing, China). The peanut hull and straw biochar were labeled as PBC and PSBC, respectively. Before the experiment, the initial properties of the topsoil (0–20 cm) and the biochar were evaluated (Table 2). Nan et al. provided a reference for the amount of biochar and fertilizer application [52]. The amount of biochar applied was 5% of the soil weight. In all treatments, urea, P_2_O_5_, and K_2_O was applied uniformly to each pot; the addition amounts were N 120 mg, P 15 mg, K 33 mg per pot, respectively. For the treatments that combined the application of biochar and compound fertilizer, the biochar and compound fertilizer were ground into powder and completely mixed, and then blended with potting soil.

### 4.2. Plant Sampling and Yield Measurement

At the mature stage of rice, three potted plants with consistent growth were randomly selected from each of the three treatments, numbered CK_1–3, PBC_1–3, and PSBC_1–3, respectively. After being harvested, threshed, and dried, the yield per pot of each treatment was than calculated.

### 4.3. Soil Sampling and Determination

At the mature stage, a 5-point sampling method was used to collect topsoil (0–20 cm) samples. Visible plant roots and organic residues were removed to dry the sample, and the soil chemical parameters (soil pH, NO_3_^−^–N, NH_4_^+^–N, AP, AK, and OM) were determined. The soil pH was determined via the glass electrode method. An AA3–A001–02E continuous flow injection analyzer (Bran–Luebbe, Norderstedt, Germany) was adopted to determine the contents of NO_3_^−^–N and NH_4_^+^–N in the soil [53]. The potassium dichromate volumetric method combined with the external heating method was adopted to measure the OM of the soil. The ammonium acetate extraction–flame photometric method and the sodium bicarbonate extraction–molybdenum-antimony colorimetric method were utilized to determine the AK and AP, respectively [54].

### 4.4. Soil Metagenomic DNA Extraction, Sequencing, and Data Analysis

Topsoil (0–20 cm) samples were collected from each treatment using the 5-point sampling method and were evenly mixed [55]. The soil samples were divided into sterilized centrifuge tubes (15 mL) using the quartering method, stored at a low temperature with liquid nitrogen [56], and transported to the laboratory after about 5 h for subsequent metagenomic analysis. The metagenomic sequencing was completed by Majorbio Pharm Technology, Co., Ltd., Shanghai, China. All samples with a quality inspection result of B or above met the requirements for standard database construction, which could proceed with subsequent steps. The detailed metagenomic sequencing and analysis process are described in the Supplementary Material, including DNA extraction and sequencing, data quality control and splicing assembly, non-redundant gene-set construction, species and functional annotation, and bioinformatics analysis [57,58,59,60,61,62,63,64,65,66,67,68].

### 4.5. Statistical Analyses and Bioinformatics

Data obtained from the soil and biochar analyses were used for variance analysis with the SPSS 20.0 software (IBM Corp, Armonk, NY, USA); graphs were drawn in Origin 8.0 (Origin Lab Corporation, Northampton, MA, USA) [69]. The bioinformatics data were analyzed using the R statistical software package in RStudio version 0.99.446 (RStudio, Inc., Boston, MA, USA, 2015). The detailed bioinformatics analysis methods and tools are described in the Supplementary Materials [56,70,71,72,73,74,75,76].

## 5. Conclusions

From the above results and discussions, it can be seen that high-dose biochar application (5%, *w*/*w*) has a significant chemical toxicity on rice production, especially with the spike number per pot and the percentage of setting. Peanut hulls, with a loose texture and pore structure, are a raw material with stronger effects for preparing biochar than peanut straw in terms of its physical structure. Although high-dose biochar application had no significant effect on the soil fungi, it decreased the diversity and stability of the soil bacterial and archaeal communities. The results also showed that biochar was highly involved in soil N- and C-cycling in the rice monoculture system. By changing the soil carbon/nitrogen ratio, high-dose biochar affected the nutrient metabolism and cycling processes of the soil microorganisms, including inhibiting the potential of the ammonia oxidation, nitrification, and denitrification processes, promoting the DNRA process, and increasing the abundance of related genes involved in carbohydrate enzymes. In the context of the current world food crisis, although biochar addition has a potential role in the regulation of soil N-, C-, and P-cycling, it should not be blindly used as an alternative strategy to inorganic fertilizer to achieve soil improvement and sustainable land production.

## Figures and Tables

**Table 1:** Soil chemical properties of different treatments.

Treatment	CK	PBC	PSBC
pH	6.93 ± 0.01 b	6.97 ± 0.02 a	6.98 ± 0.03 a
NO_3_^−^–N (mg kg^−1^)	9.83 ± 0.15 a	7.60 ± 0.10 c	8.97 ± 0.15 b
NH_4_^+^–N (mg kg^−1^)	10.77 ± 0.40 b	13.67 ± 0.54 a	10.24 ± 0.42 b
AP (mg kg^−1^)	30.16 ± 1.65 b	28.52 ± 1.35 b	32.86 ± 0.85 a
AK (mg kg^−1^)	27.00 ± 0.56 c	38.16 ± 1.92 b	50.82 ± 1.96 a
OM (g kg^−1^)	40.75 ± 0.46 c	49.77 ± 0.79 b	72.97 ± 1.17 a

Means are followed by ± standard deviations. Different lowercase letters mean significant differences based on a one-way ANOVA followed by Duncan’s multiple-range tests (*p* < 0.05). CK: control; PBC: peanut hull biochar; PSBC: peanut straw biochar. NO_3_^−^–N: nitrate nitrogen; NH_4_^+^–N: ammonium nitrogen; AP: available phosphorus; AK: available potassium; OM: organic matter.

**Table 2:** Primary properties of topsoil (0–20 cm) and the tested biochar.

Parameters	Topsoil	Biochar	
		PBC	PSBC
pH	6.38	10.87	11.69
NO_3_^−^–N (mg kg^−1^)	5.1	3.42	3.64
NH_4_^+^–N (mg kg^−1^)	43	3.93	3.72
AP (mg kg^−1^)	63.2	/	/
AK (mg kg^−1^)	118.37	/	/
OM (g kg^−1^)	8.64	/	/
Specific surface area (m^2^ g^−1^)	/	166.70	128.53
Productivity (%)	/	38.77	41.37
Ashes (%)	/	21.64	23.85

**Table 3:** Rice yield and yield components of different treatments.

Treatment	CK	PBC	PSBC
Spike (pot^−1^)	53.00 ± 1.00 a	45.67 ± 2.52 b	49.00 ± 3.61 ab
Grain (spike^−1^)	82.00 ± 3.61 a	85.33 ± 2.08 a	87.33 ± 2.08 a
Thousand-grain weight (g)	26.17 ± 0.43 a	25.42 ± 0.19 b	25.88 ± 0.24 ab
Percentage of setting (%)	0.903 ± 0.021 a	0.817 ± 0.015 b	0.900 ± 0.010 a
Yield (g pot^−1^)	102.66 ± 2.96 a	81.06 ± 8.01 b	99.76 ± 9.22 ab

Different lowercase letters mean significant differences based on a one-way ANOVA followed by Duncan’s multiple-range tests (*p* < 0.05).

## Data Availability

The data files (read in FASTQ format) were deposited at the NCBI SRA database under the BioProject, accession No. PRJNA997742 (https://www.ncbi.nlm.nih.gov/sra/PRJNA997742 (accessed on 24 July 2023)).

## Supplementary Materials

The following supporting information can be downloaded at https://www.mdpi.com/article/10.3390/ijms242015043/s1.
