# Supplementary material for: The Inhibiting Effects of High-Dose Biochar Application on Soil Microbial Metagenomics and Rice (Oryza sativa L.) Production
Source: Int J Mol Sci. 2023 Oct 10;24(20):15043. doi: 10.3390/ijms242015043 (PMC10606461; doi:10.3390/ijms242015043)
Supplement: Supplementary file 1 [file ijms-24-15043-s001.zip › ijms-2644532-supplementary.pdf]

Supplementary Material

1. Metagenome Sequencing and Analysis Process

1.1. DNA Extraction and Sequencing

According to the manufacturer's instructions, the total DNA of the soil microbial community was extracted from 0.25 g (total wet weight) of soil using a soil DNA extraction kit (E.Z.N.A.® Soil DNA Kit, Omega), and each soil sample was extracted three times [57]. For the quality inspection of samples, TBS-380, NanoDrop2000, and 1% agarose gel electrophoresis were adopted to detect the concentration, purity, and integrity of the DNA, respectively. The results of all samples were above grade B, which meets the requirements of standard library construction. Therefore, subsequent steps could be carried out. The DNA was fragmented by a gene company (Covaris M220, China), and fragments interrupted by about 400 bp were screened. A NEXTFLEX Rapid DNA-Seq (Bio Scientific, USA) library construction kit was utilized to build a PE library [58]. After bridge-PCR amplification, metagenome sequencing was carried out using an Illumina NovaSeq6000 (Illumina, USA) sequencing platform [59].

1.2. Data Quality Control and Splicing Assembly

Fastp v0.20.0 (https://github.com/OpenGene/fastp) was used for the quality control of the original data [60]. BWA was adopted to compare reads to the DNA sequence of the host, and contaminated reads with a high comparison similarity were removed. The splicing assembly of the optimized sequence was conducted by utilizing Megahit V1.1.2 (https://github.com/voutcn/megahit) based on the principle of succinct de Bruijn graphs. Contigs (≥ 300 bp) were selected as the final assembly results in the splicing results [61].

1.3. Non-redundant Gene Set Construction

CD-HIT v4.6.1 (http://www.bioinformatics.org/cd-hit/) was utilized to cluster the predicted gene sequences of all samples (default parameters: 90% identity, 90% coverage) [62]. For each sample, the longest gene was taken as the representative sequence to construct a non-redundant gene set. SOAPAligner v.soap 2.21release (https://github.com/ShujiaHuang/SOAPaligner) was adopted to compare the high-quality reads with the non-redundant gene set of each sample (default parameter: 95% identity) [63-64], and the abundance information of genes in the corresponding sample was counted. Prodigal V2.6.3 (https://github.com/hyattpd/Prodigal) was used to predict the ORF of the assembled contigs [65], and genes with s nucleic acid length greater than or equal to 100 bp were chosen and translated into amino acid sequences, and a statistical table of the gene prediction results of each sample was obtained.

1.4. Species and Functions Annotation

DIAMOND (https://github.com/bbuchfink/diamond) was used to compare the non-redundant gene set with the NR database (https://ftp.ncbi.nlm.nih.gov/blast/db/FASTA/) (parameter: blastp; E-value ≤ 1e-5), and species annotation information at the various taxonomic levels of kingdom, phylum, class, order, family, genus, and species was obtained [66-67]. "*Taxonomy level abbreviation_name*" was selected as the annotation method for the microbial species. If the species could not be identified at the current taxonomy level, it was annotated as "*taxonomy level abbreviation_unclassified_abbreviation of the lowest taxonomic level that can be recognized_name*". The abbreviations of the taxonomy level are *k* (kingdom), *p* (phylum), *c* (class), *o* (order), *f* (family), *g* (genus), and *s* (species).

DIAMOND was adopted to compare the sequence of the non-redundant gene set with the gene database of KEGG (http://www.genome.jp/kegg/) to obtain its functional information corresponding to the gene. The sum of the gene abundance corresponding to the KO, Pathway, EC, and Module was used to calculate the abundance of corresponding functional categories [68].

1.5. Bioinformatics analyses

Bioinformatics data were analyzed using the R statistical software package in RStudio version 0.99.446 (RStudio, Inc. 2015). Microbial relative abundance was calculated using the RPKM (reads per kilobase million) method [70]. The richness (Chaoindex) and diversity (Shannon and Simpson index) indices of the microbial species were used to examine the α diversity, and were displayed using the R statistical software [71]. The β

diversity was estimated using the principal component analysis (PCA) [72], and a permutational multivariate analysis of variance (PERMANOVA) based on the Bray–Curtis distance was adopted to investigate the composition differences in the microbial communities among the various samples [73]. The correspondence between the relative abundance of microbial communities at the class level in the different samples was visualized using Circos-0.67-7 (http://circos.ca/ (accessed on 10 March 2023)). The characterization of the microbial communities in each treatment was performed via the linear discriminant analysis effect size (LEfSe) (http://huttenhower.sph.harvard. edu/ galaxy/ root? tool_ id=lefse_upload (accessed on 12 March 2023)) for the biomarker discovery and explanation, which emphasized the different microorganisms among the groups [74-75]. The soil chemical properties were regarded as environmental factors, and a redundancy analysis (RDA) (RPKM > 1%) was employed to assess the associations between the soil environmental factors and archaea [76]. A correlation network between a soil biochemical property and a community composition of enriched soil bacteria and archaea at the genus level was considered statistically significant if Spearman's correlation coefficient (r) was > 0.7 and the p_value was < 0.05 [56]. The "Networkx" package was employed to assess the associations between the soil variables and the bacterial community composition. KEGG Orthology (KO) and KEGG level 2 terms were used to investigate the function genes of the soil microbial community in this study. To explore the significant differences between the functional microbes, a Kruskal−Wallis H test was conducted for the nonparametric testing of three independent samples. Then, a post hoc test based on Tukey−Kramer was also implemented to compare the selected groups in pairs.

References

57. Schmidt, R.; Mitchell, J.; Scow, K. Cover cropping and no-till increase diversity and symbiotroph: saprotroph ratios of soil fungal communities. *Soil Biol. Biochem.* **2019**, *129*, 99–109. https://doi.org/ 10.1016/j.soilbio.2018.11.010.
58. Wu, L.Y.; Wen, C.Q.; Qin, Y.H.; Yin, H.Q.; Tu, Q.C.; Joy, D.V.N.; Yuan, T.; Yuan, M.T.; Deng, Y.; Zhou, J.Z. Phasing amplicon sequencing on illumina miseq for robust environmental microbial community analysis. *Bmc Microbiol*. **2015**, *15*, 125. https://doi.org/10.1186/s12866-015-0450-4.
59. Mueller, R.C.; Paula, F.S.; Mirza, B.S.; Rodrigues, J.L.M.; Nüsslein, K.; Bohannan, B.J.M. Links between plant and fungal communities across a deforestation chronosequence in the amazon rainforest. *ISME J*. **2014**, *8*, 1551-1551. https://doi.org/ 10.1038/ismej.2014.62.
60. Bolger, A.M.; Marc, L.; Bjoern, U. Trimmomatic: a flexible trimmer for illumina sequence data. *Bioinformatics* **2014**, *30*, 2114-2120. https://doi.org/10.1093/bioinformatics/btu170.
61. Li, D. H.; Liu, C.M.; Luo, R.B.; Kunihiko, S.; Tak-Wah, L. Megahit: an ultra-fast single-node solution for large and complex metagenomics assembly via succinct de brujn graph. *Bioinformatics* **2015**, *31*, 1674-1676. https://doi.org/10.1093/bioinformatics/btv033.
62. Fu, L. M.; Niu, B.F.; Zhu, Z.W.; Wu, S.T.; Li, W.Z. Cd-hit: accelerated for clustering the next-generation sequencing data. *Bioinformatics* **2012**, *28*, 3150-3152. https://doi.org/10.1093/bioinformatics/bts565.
63. Luo, R.B.; Liu, B.H.; Xie, Y.L.; Li, Z.Y.; Huang, W.H.; Yuan, J.Y.; He, G.Z., Chen, Y.X.; Pan, Q.; Liu, Y.J.; et al. Erratum: soapdenovo2: an empirically improved memory-efficient short-read de novo assembler. *GigaScience* **2015**, *4*, 1. https://doi.org/ 10.1186/s13742-015-0069-2.
64. Shi, P.; Jia, S.Y.; Zhang, X.X.; Tong, Z.; Cheng, S.P.; Li, A.M. Metagenomic insights into chlorination effects on microbial antibiotic resistance in drinking water. *Water Res.* **2013**, *47*, 111-120. https://doi.org/ 10.1016/j.watres.2012.09.046.
65. Hyatt, D.; Chen, G.L.; Locascio, P.F.; Land, M.L.; Larimer, F.W.; Hauser, L.J. Prodigal: prokaryotic gene recognition and translation initiation site identification. *Bmc Bioinformatics* **2010**, *11*, 119. https://doi.org/ 10.1186/1471-2105-11-119.
66. Buchfink, B.; Xie, C.; Huson, D.H. Fast and sensitive protein alignment using DIAMOND. *Nat. Methods* **2015**, *12*, 59-60. https://doi.org/ 10.1038/nmeth.3176.
67. Buchfink, B.; Reuter, K.; Drost, HG. Sensitive protein alignments at tree-of-life scale using DIAMOND. *Nat. Methods* **2021**, *18*, 366–368. https://doi.org/10.1038/s41592-021-01101-x.
68. Kanehisa, M.; Goto, S.; Kawashima, S.; Okuno, Y.; Hattori, M. The KEGG resource for deciphering the genome. *Nucleic Acids Res*. **2004**, *32*, 277–280. https://doi.org/ 10.1093/nar/gkh063.
70. Lawson, C.E.; Wu, S.; Bhattacharjee, A.S.; Hamilton, J.J.; McMahon, K.D.; Goel, R.; Noguera, D.R. Metabolic network analysis reveals microbial community interactions in anammox granules. *Nat. Commun.* **2017**, *8*, 15416. https://doi.org/ 10.1038/ncomms15416.
71. Li, P. F.; Liu, J.; Jiang, C.Y.; Wu, M.; Liu, M.; Li, Z.P. Distinct successions of common and rare bacteria in soil under humic acid amendment – A microcosm study. *Front. Microbiol.* **2019**, *10*, 2271. https://doi.org/ 10.3389/fmicb.2019.02271.

72. Stegen, J.C.; Lin, X.J.; Konopka, A.E.; Fredrickson, J.K. Stochastic and deterministic assembly processes in subsurface microbial communities. *ISME J.* **2012**, *6*, 1653–1664. https://doi.org/ 10.1038/ismej.2012.22.
73. Rivas, M.N.; Burton, O.T.; Wise, P.; Zhang, Y.Q.; Hobson, S.A.; Lloret, M.G.; Chehoud, C.; Kuczynski, J.; DeSantis, T.; Warrington, J.; et al. A microbiota signature associated with experimental food allergy promotes allergic sensitization and anaphylaxis. *J. Allergy. Clin. Immun.* **2013**, *131*, 201-212. https://doi.org/ 10.1016/j.jaci.2012.10.026.
74. Segata, N.; Izard, J.; Waldron, L.; Gevers, D.; Miropolsky, L.; Garrett, W.S.; Huttenhower, C. Metagenomic biomarker discovery and explanation. *Genome Biol.* **2011**, *12*, R60. https://doi.org/ 10.1186/gb-2011-12-6-r60.
75. Zhang, C.H.; Li, S.F.; Yang, L.; Huang, P.; Li, W.J.; Wang, S.Y.; Zhao, G.P.; Zhang, M.H.; Pang, X.Y.; Yan, Z.; Liu, Y.; Zhao, L.P. Structural modulation of gut microbiota in life-long calorie-restricted mice. *Nat. Commun.* **2013**, *4*, 2163. https://doi.org/ 10.1038/ncomms3163.
76. Goethem, M.W.V.; Pierneef, R.; Bezuidt, O.K.I.; Peer, Y.V.D.; Cowan, D.A.; Makhalanyane, T.P. A reservoir of 'historical' antibiotic resistance genes in remote pristine antarctic soils. *Microbiome.* **2018**, *6*, 40. https://doi.org/ 10.1186/s40168-018-0424-5.

2. Supplementary Figures

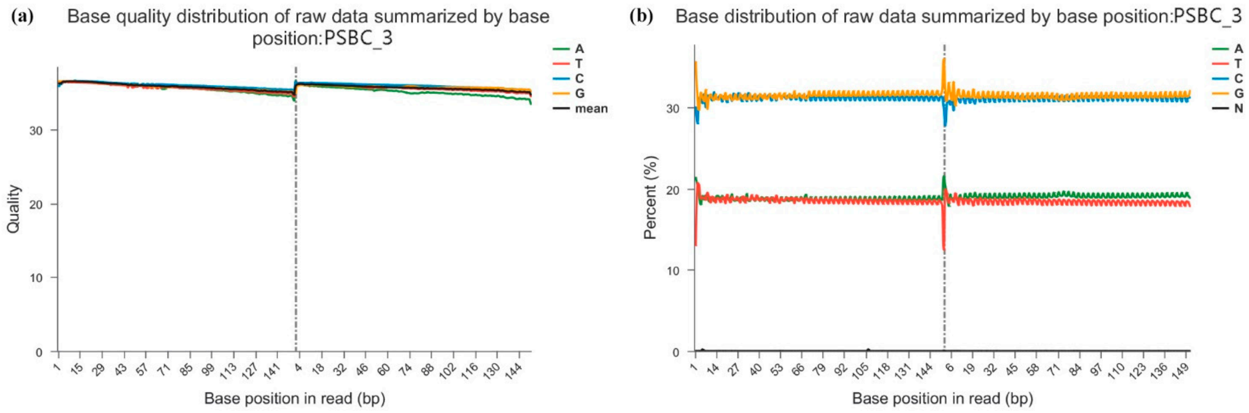

Figure S1. (a) Base distribution of raw data summarized based on base position: PSBC_3. The abscissa is the base position coordinate of the reads, and the ordinate is the base quality of the reads. In this figure, the range specified by the vertical "I" is the distribution interval of the base quality of all reads, the vertical square is the interquartile value range of quality, and the thick line is the median of the quality value. The left side of the dotted line shows the base mass of read1, and the right side shows the base mass of read2. (b) Base quality distribution of raw data summarized based on base position: PSBC_3. The abscissa is the base coordinate of the reads, and the ordinate is the percentage of bases A, C, G, T, and N of all reads at the sequencing position (such as the first sequencing base). Different bases are represented by different colors.

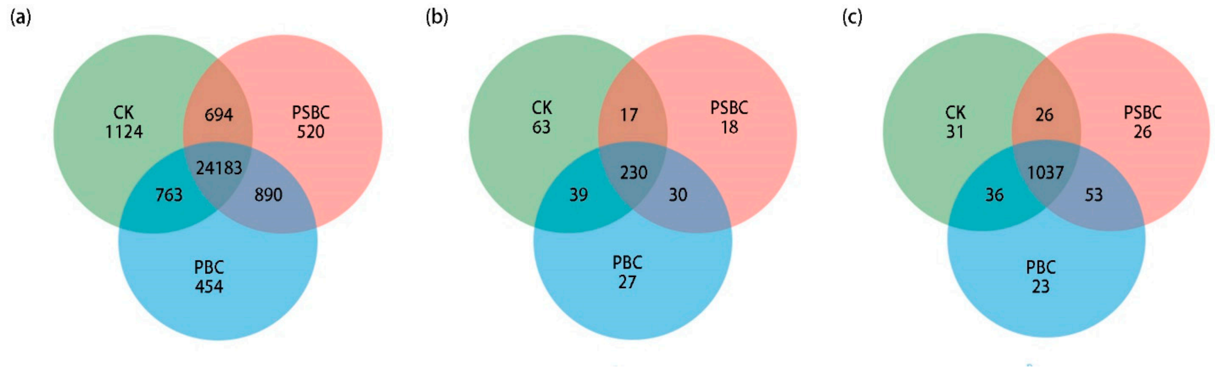

Figure S2. Venn diagrams of (a) bacterial, (b) fungal, and (c) archaeal communities at the species level.

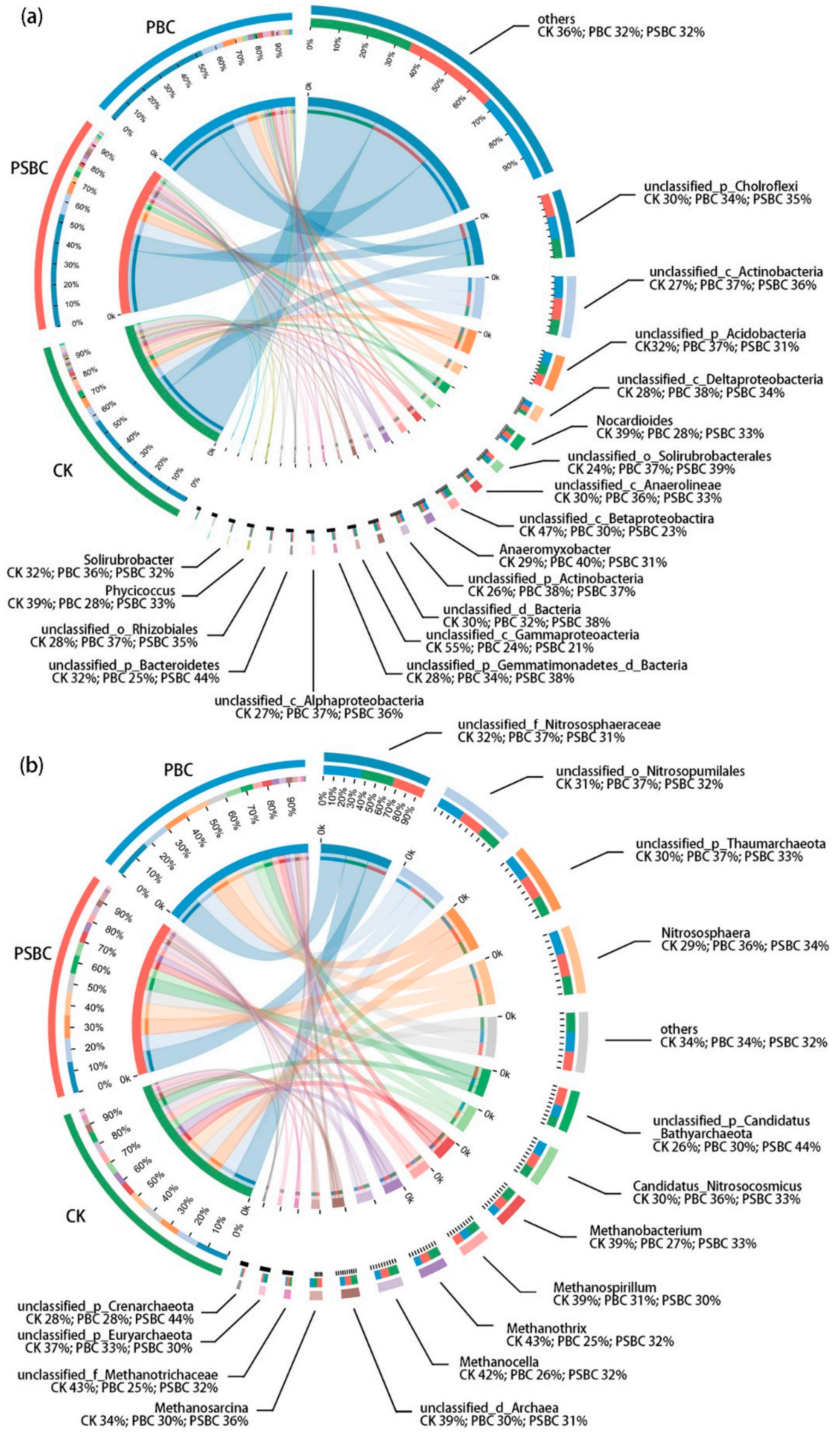

Figure S3. Circos diagram of (A) soil bacterial and (B) archaeal communities at the genus level. The left semicircle represents the species abundance composition of the samples, and the right semicircle represents the distribution ratio of species in different samples at the genus level. The genera with RPKM < 1% are merged into the others in this figure.

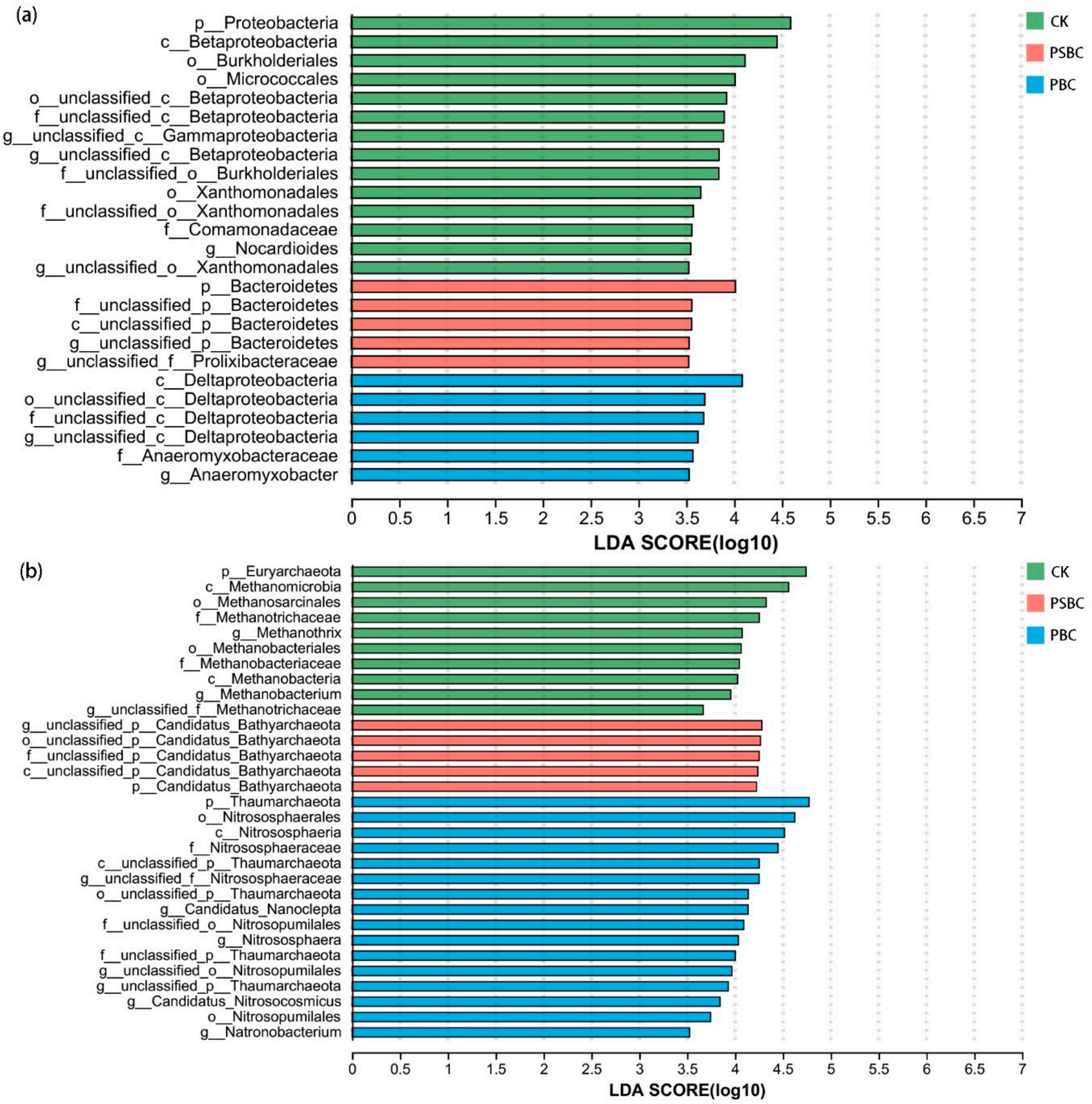

Figure S4. The linear discriminant analysis (LDA) score of soil (a) bacterial and (b) archaeal communities. The comparison policy was set to all-against-all and the threshold value was set to 3.5.

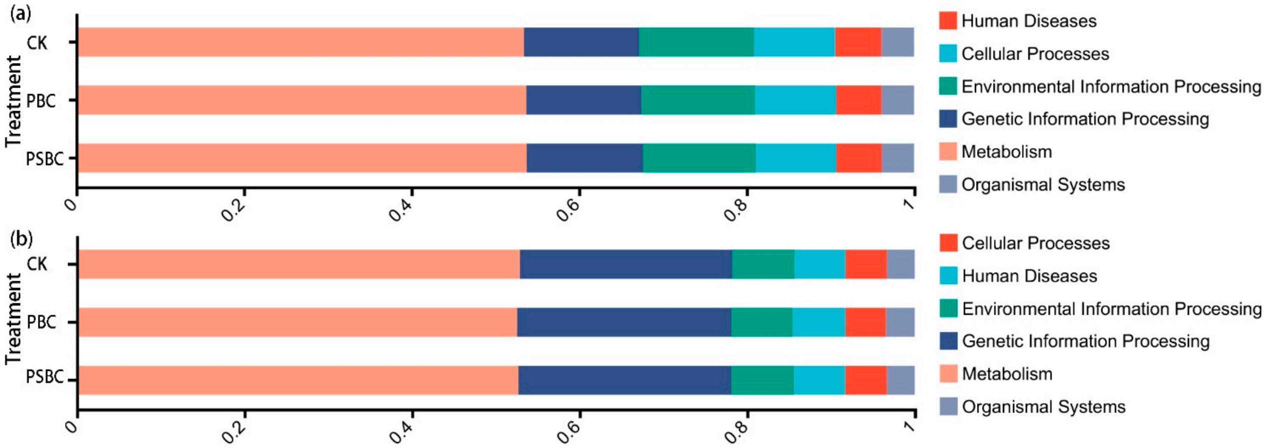

Figure S5. Influence of high-dose biochar application on the relative abundance of soil (A) bacterial and (B) archaeal functional composition at KEGG_L1.

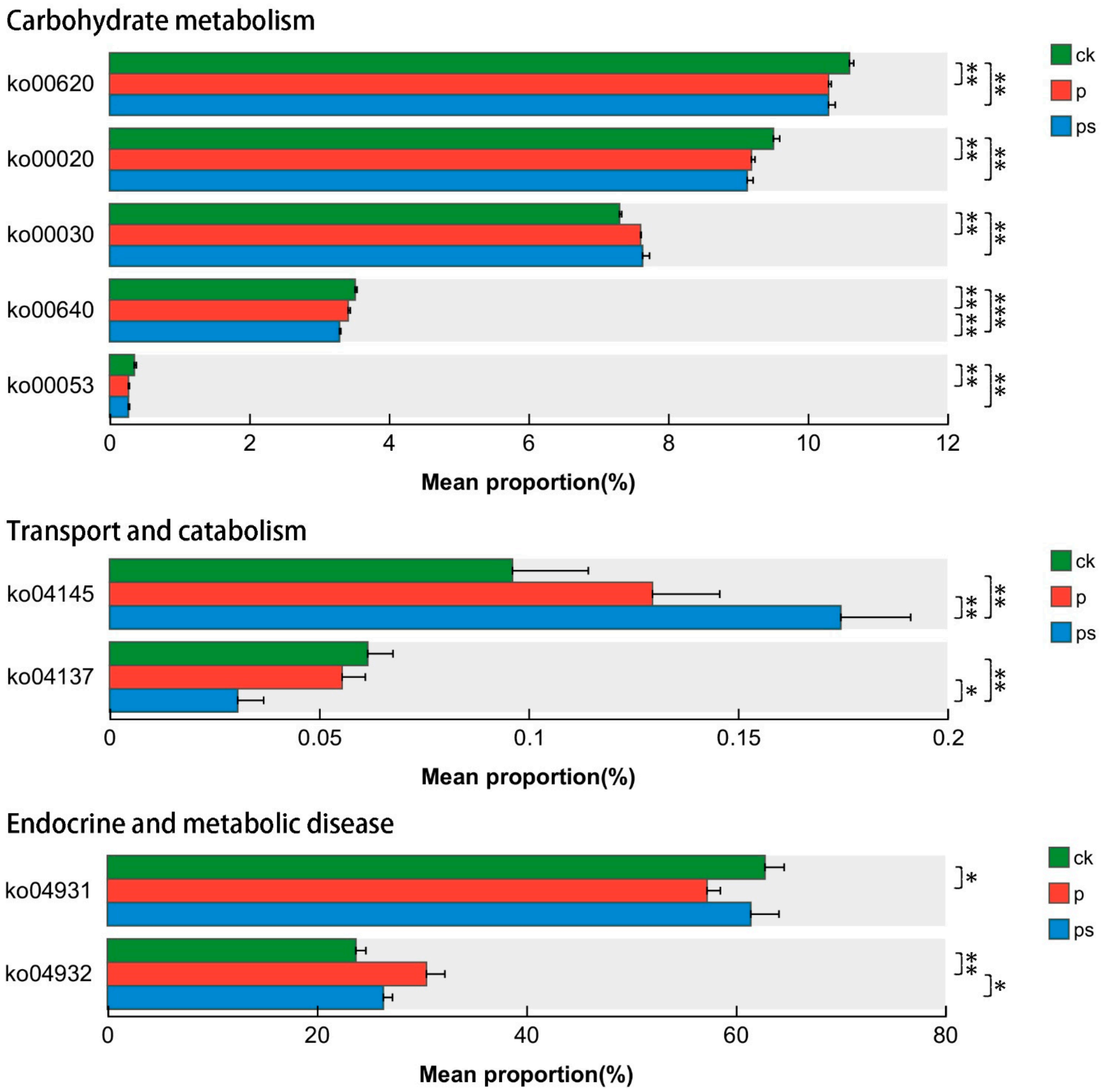

Figure S6. Kruskal–Wallis H test bar plot of functional composition at KEGG_L3.

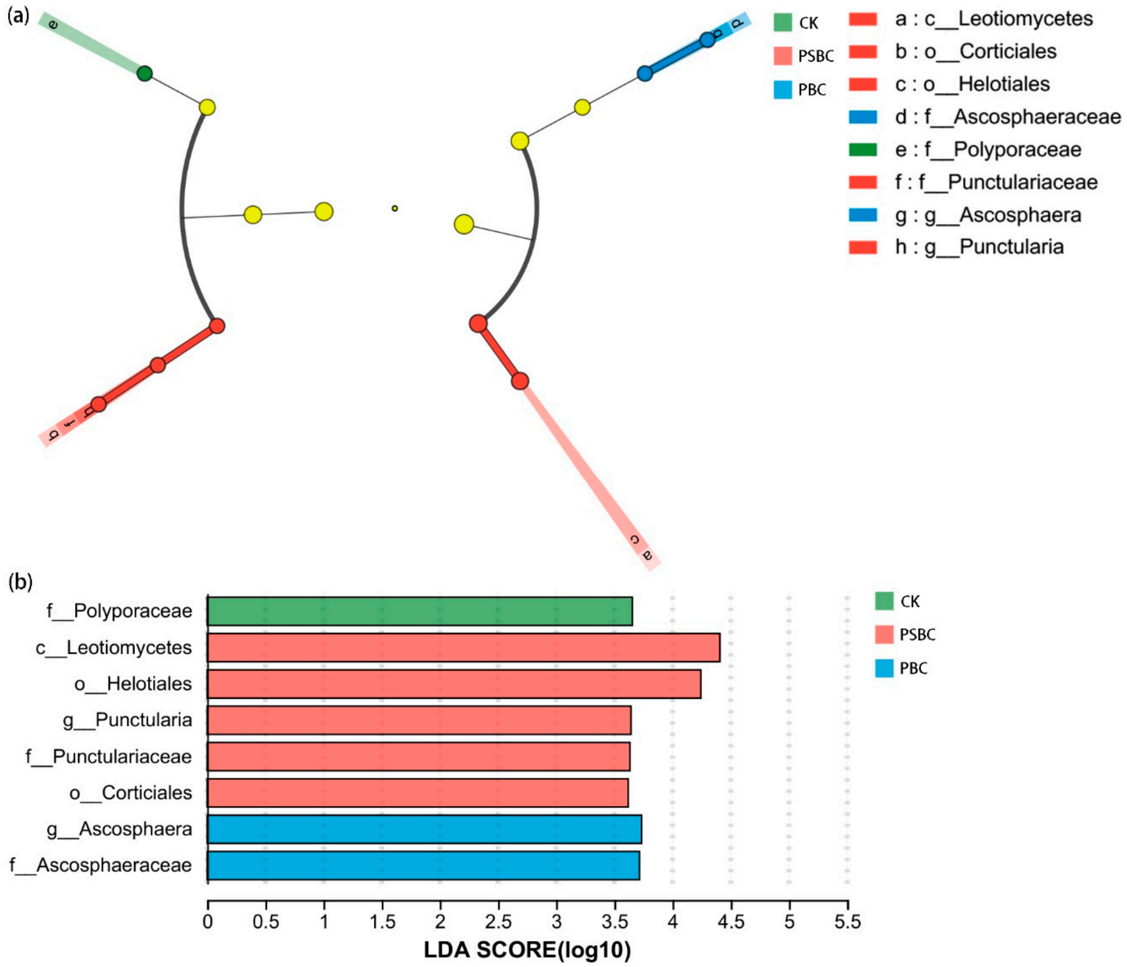

Figure S7. (a) Cladogram of soil fungal communities. The colored dots represent the microbial groups that are significantly enriched in the corresponding group, and from the center outward, they represent the phylum, class, order, family, and genus levels. The colored shadows represent trends of the significantly differed taxa. Yellow nodes represent microbial taxa that have no significant effect on differences between groups. (b) LDA score of each colored dot (LDA > 3.5).
